# Efficacy and safety of reduced-dose valganciclovir prophylaxis for cytomegalovirus infection after pediatric kidney transplantation

**DOI:** 10.1007/s10157-026-02876-z

**Published:** 2026-05-13

**Authors:** Ryo Nakatani, Yoko Shirai, Taro Ando, Yoko Yamasaki, Yohei Kume, Koji Tsugawa, Kohei Unagami, Tomokazu Shimizu, Hideki Ishida, Kenichiro Miura

**Affiliations:** 1 Department of Pediatric Nephrology, Tokyo Women’s Medical University, 8-1, Kawada-cho, Shinjuku-ku, Tokyo, Japan; 2 Department of Pediatrics, Gunma University Graduate School of Medicine, Gunma, Japan; 3 Department of Pediatrics, School of Medicine, Fukushima Medical University, Fukushima, Japan; 4 Department of Pediatrics, Hirosaki University Hospital, Aomori, Japan; 5 Department of Organ Transplant Medicine, Tokyo Women’s Medical University, Tokyo, Japan

**Keywords:** Kidney transplantation, Children, Cytomegalovirus, Valganciclovir, Neutropenia

## Abstract

**Background:**

The optimal dosing strategy for valganciclovir (VGCV) prophylaxis against cytomegalovirus (CMV) infection in pediatric patients after kidney transplantation (KT) remains uncertain because of the narrow therapeutic window between antiviral efficacy and safety.

**Methods:**

This study included pediatric patients who received VGCV prophylaxis after KT at a single center between December 2023 and December 2024. VGCV was administered once daily for 200 days using a reduced-dose regimen. Approximately 50% of the U.S. Food and Drug Administration–recommended dose was used for high-risk patients defined as donor-positive and recipient-negative, and 33% for intermediate-risk patients defined as recipient-positive. The incidence of CMV infection within 200 days after KT and VGCV-related adverse events (AEs) were evaluated.

**Results:**

Nine patients with a median age of 8 years were included, with a median observational period of 431 days after KT. CMV infection within 200 days occurred in four patients, all of whom were high-risk patients. No CMV disease occurred during the observational period. Neutropenia occurred in six patients, including five high-risk patients and one intermediate-risk patient. Three high-risk patients developed febrile neutropenia. VGCV dose reduction and/or discontinuation due to hematologic AEs was required in four patients.

**Conclusions:**

The reduced-dose VGCV prophylaxis regimen was associated with a high incidence of CMV infection and AEs in high-risk patients, indicating a suboptimal balance between efficacy and safety. In intermediate-risk patients, the regimen appeared effective and tolerable. Further optimization of prophylactic strategies is required, particularly for high-risk pediatric recipients.

**Supplementary Information:**

The online version contains supplementary material available at 10.1007/s10157-026-02876-z.

## Introduction

Cytomegalovirus (CMV) is a major pathogen that adversely affects graft function and long-term outcomes after kidney transplantation (KT) [1]. Based on donor and recipient CMV serostatus, the risk of CMV infection is stratified into high (donor-positive/recipient-negative, D + /R −), intermediate (recipient-positive, R +), and low (donor-negative/recipient-negative, D − /R −) categories. Given the high incidence of CMV infection in the high-risk group, appropriate strategies for the prevention and management of CMV infection after KT are essential [2].

Pediatric patients after KT are frequently CMV-seronegative prior to KT, placing them at increased risk of post-transplant CMV infection compared with adult recipients [3, 4]. Consequently, CMV infection represents a major clinical concern in pediatric KT. According to international consensus guidelines, both prophylaxis and preemptive therapy are recommended as acceptable management strategies for pediatric patients after KT at high and intermediate risk, largely extrapolated from adult practice [5]. However, the optimal dosing strategy of valganciclovir (VGCV), the most commonly used agent for CMV prophylaxis, has not been clearly defined in Japanese pediatric patients. In addition, differences in clinical practice patterns and susceptibility to hematologic toxicity reported in Japanese pediatric patients after KT may influence the risk–benefit balance of VGCV prophylaxis compared with international cohorts [6–9].

At our institution, we have conducted a stepwise series of dose-optimization studies evaluating VGCV prophylaxis after pediatric KT [6–9]. These studies were conducted during sequential institutional protocol periods (August 2018 to March 2019 [first study] [6], June 2020 to January 2022 [second study] [7], January to June 2022 [third study] [8], and June 2022 to December 2023 [fourth study] [9]), using broadly similar eligibility criteria but different VGCV dosing regimens and surveillance strategies for CMV infection, each reflecting stepwise modifications based on the findings of the preceding study. In the first and second studies, VGCV administered at relatively higher exposure levels (51–90% and 26–67% of the recommended dose) was associated with frequent hematologic adverse events (AEs) despite adequate antiviral efficacy [6, 7]. Subsequent attempts to reduce toxicity using lower-dose regimens (25% and 33% of the recommended dose in the third and fourth studies, respectively) resulted in insufficient efficacy in high-risk patients, with frequent CMV infection occurring within 200 days after KT [8, 9], whereas intermediate-risk patients demonstrated acceptable outcomes at 33% dosing [9].

Given these sequential findings and the need to refine dosing primarily in high-risk recipients, the present study therefore evaluated the efficacy and safety of VGCV prophylaxis after KT in Japanese pediatric patients under an updated regimen corresponding to 50% of the recommended VGCV dose in high-risk patients, while maintaining the previously effective 33% regimen in intermediate-risk patients. Because 33% dosing had demonstrated acceptable efficacy and safety in intermediate-risk recipients in our previous study [9], the primary modification of the present protocol focused on high-risk recipients.

## Methods

### Study participants

This study included pediatric patients younger than 20 years of age who underwent KT at Tokyo Women’s Medical University between December 2023 and December 2024 and were managed with VGCV prophylaxis. Clinical data were obtained from electronic medical records.

The present study was conducted during a distinct institutional protocol period reflecting stepwise evolution of VGCV dosing strategies. The cohort analyzed in this study did not overlap with patient populations included in our previous dose-optimization studies [6–9].

### CMV prophylaxis regimen

VGCV prophylaxis was initiated on postoperative day 10 and continued for 200 days. VGCV was administered once daily as an oral suspension to facilitate accurate dose adjustment.

The daily VGCV dose was calculated using the U.S. Food and Drug Administration–recommended pediatric dosing formula: 7 × estimated glomerular filtration rate (eGFR; mL/min/1.73 m^2^) × body surface area (m^2^), expressed in milligrams [10]. Based on our previous institutional experience, which demonstrated a narrow therapeutic window of VGCV in pediatric patients, a reduced-dose strategy was adopted to balance antiviral efficacy and safety. Specifically, patients classified as high risk for CMV infection received approximately 50% of the recommended VGCV dose, whereas those classified as intermediate risk received approximately 33%.

### CMV monitoring and definitions

CMV monitoring was performed using the pp65 antigenemia assay and quantitative nucleic acid testing, in accordance with routine clinical practice at our institution. CMV infection was defined as evidence of CMV antigen detection or nucleic acid testing in blood, regardless of the presence of symptoms, in accordance with international consensus guidelines for the management of CMV in solid-organ transplant recipients [5]. CMV disease was defined as CMV infection accompanied by clinical symptoms or signs, including CMV syndrome or end-organ disease, according to the same guidelines [5]. The primary efficacy outcome was the occurrence of CMV infection and CMV disease within 200 days after KT. Secondary outcomes included seroconversion following CMV infection. In our institutional practice, CMV surveillance intensity differed according to CMV risk status. During the first month after KT, monitoring was typically performed two to three times per week in high-risk recipients and once to twice per week in intermediate-risk recipients. Thereafter, testing intervals were generally every 1–2 weeks in high-risk patients and every 2–4 weeks in intermediate-risk patients, although adjustments were made according to clinical conditions.

### Treatment of CMV infection

CMV infection was initially treated with ganciclovir (GCV), followed by VGCV when tolerated. In high-risk patients, GCV therapy was initiated when the pp65 antigenemia assay detected ≥ 1 positive cell per 200,000 neutrophils. In intermediate-risk patients, GCV was initiated when the pp65 antigenemia assay showed ≥ 10 positive cells per 200,000 neutrophils [11] or when the CMV viral load reached approximately ≥ 1,000 IU/mL [12].

### Safety assessment

Safety was evaluated based on the incidence of VGCV-related AEs. Severe neutropenia was defined as an absolute neutrophil count (ANC) < 500/µL, and profound neutropenia as < 100/µL [13]. Febrile neutropenia (FN) was defined as a sustained body temperature ≥ 38.0 °C with an ANC < 500/µL, or an expected decline to < 500/µL within 48 h, in accordance with established criteria [13].

VGCV dose reduction was performed when the ANC decreased to approximately < 1,500/µL, and granulocyte colony-stimulating factor was administered as needed. VGCV prophylaxis was discontinued upon severe neutropenia or the development of FN. Re-initiation of VGCV after discontinuation was determined at the discretion of the attending physician.

Because post-kidney transplant cytopenias may be multifactorial, concomitant medications including mycophenolate mofetil and prophylactic agents such as trimethoprim–sulfamethoxazole were reviewed during clinical management. Dose adjustments or temporary reduction of these agents were performed at the discretion of the attending physician when clinically indicated; however, a formal quantitative analysis of their independent contribution to cytopenias was not performed in this study.

### Kidney function and immunosuppression

eGFR was calculated using a creatinine-based equation validated for Japanese children and adolescents with chronic kidney disease [14]. For patients aged 18 years or older at the time of KT, the Japanese adult eGFR equation was applied [15]. Body surface area was calculated using the Mosteller formula [16].

All patients received induction and maintenance immunosuppressive therapy consisting of tacrolimus, mycophenolate mofetil, methylprednisolone, and basiliximab. Tacrolimus was initiated at a dose of 0.15 mg/kg, with the dose adjusted to achieve target trough concentrations of 10.0 ng/mL during the first month after KT, 7.0 ng/mL during months 2–5, and 5.0 ng/mL from 6 months after KT onward. Mycophenolate mofetil was initiated at a dose of 500 mg/m^2^ and increased to 800 mg/m^2^ on postoperative day 1. Methylprednisolone was administered at an initial dose of 15 mg/kg, followed by gradual tapering to an alternate-day dose of 4 mg by one month after KT. Basiliximab was administered intraoperatively and on postoperative day 4. Trimethoprim–sulfamethoxazole was administered two to three times weekly for prophylaxis against *Pneumocystis jirovecii* pneumonia.

## Results

### Patient characteristics

The clinical characteristics of the patients are summarized in Table 1. A total of nine pediatric patients who underwent KT were included, of whom five were male. The median age at KT was 8 years (interquartile range [IQR], 6–18 years). Three patients underwent living-donor KT, and no ABO-incompatible KTs were performed. No patients received rituximab. Five patients were classified as high-risk and four as intermediate-risk for CMV infection. The median eGFR at initiation of VGCV prophylaxis was 92.4 mL/min/1.73 m^2^ (IQR, 66.3–105.0 mL/min/1.73 m^2^).

**Table 1:** Clinical characteristics of the patients

Risk	Patient	Age at KT	Gender	BSA (m^2^)	Original kidney disease	Donor type	ABO-compatibility	Immunosuppressants	eGFR at initiating of VGCV prophylaxis (mL/min/1.73m^2^)	VGCV dose (mg)
High (D + /R-)	1	19	Female	1.34	Hypoplastic kidneys	Deceased	Compatible	TAC, MMF, BXM, methylprednisolone	68.3	300
	2	5	Female	0.63	Hypoplastic kidneys	Deceased	Compatible	TAC, MMF, BXM, methylprednisolone	91.3	150
	3	19	Male	1.55	ARPKD	Deceased	Compatible	TAC, MMF, BXM, methylprednisolone	64.2	450
	4	13	Male	1.14	Nephronophthisis	Living	Compatible	TAC, MMF, BXM, methylprednisolone	97.6	400
	5	7	Male	0.73	Reflux nephropathy	Deceased	Compatible	TAC, MMF, BXM, methylprednisolone	92.4	180
Intermediate (R +)	6	7	Female	0.60	Joubert syndrome	Deceased	Compatible	TAC, MMF, BXM, methylprednisolone	116.1	120
	7	8	Male	0.93	Nephronophthisis	Deceased	Compatible	TAC, MMF, BXM, methylprednisolone	96.2	180
	8	5	Female	0.61	Neonatal acute kidney injury	Living	Compatible	TAC, MMF, BXM, methylprednisolone	112.3	100
	9	17	Male	1.65	Hypoplastic kidneys	Living	Compatible	TAC, MMF, BXM, methylprednisolone	58.6	250

*ARPKD* Autosomal recessive polycystic kidney disease, *BSA* body surface area, *BXM* basiliximab, *CMV* cytomegalovirus, *D* donor, *eGFR* estimated glomerular filtration rate, *KT* kidney transplantation, *MMF* mycophenolate mofetil, *R* recipient, *TAC* tacrolimus, *VGCV* valganciclovir

### Prophylactic efficacy in preventing CMV infection

**Fig. 1:**
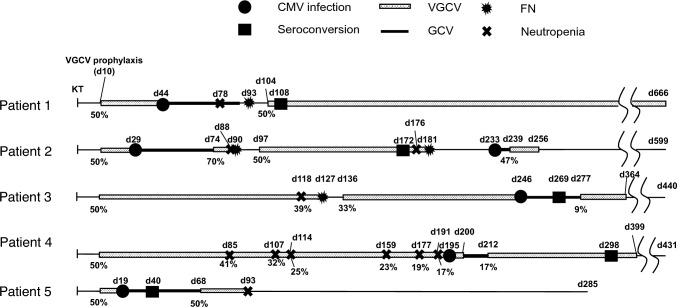
Clinical course of high-risk pediatric patients after kidney transplantation. VGCV prophylaxis was initiated on day 10. The notation “d” indicates the number of days after KT. Percentages shown in the figure represent the percentage of the recommended dose. Symbols denote the occurrence of CMV infection, seroconversion, FN, neutropenia, dose reduction or discontinuation of VGCV, and administration of ganciclovir. To ensure readability in grayscale, distinct symbols and line patterns are shown in the figure. *CMV* cytomegalovirus, *FN* febrile neutropenia, *GCV* ganciclovir, *KT* kidney transplantation, *VGCV* valganciclovir

The outcomes of VGCV prophylaxis are summarized in Table 2, and the clinical course of each high-risk patient is shown in Fig. 1. The median observational period after KT was 431 days (IQR, 330–553 days). Among high-risk patients, initial CMV infection occurred within 200 days in four of five patients. CMV infection after 200 days was observed in two patients, including one patient with recurrent infection (Table 2 and Fig. 1). Patient 2 developed CMV infection on post-transplant day 29, and was treated with GCV followed by VGCV. During antiviral treatment for CMV infection, the patient experienced two episodes of FN. Subsequently, CMV infection recurred on post-transplant day 233, which was successfully treated with GCV followed by VGCV (Fig. 1). All high-risk patients demonstrated seroconversion from CMV IgG–negative to CMV IgG-positive status. The median time from CMV infection to seroconversion was 64 days (IQR, 22–123 days).

**Table 2:** Outcomes of VGCV prophylaxis

Risk	Patient	Observational period (days)	CMV infection occurring within 200 days after KT*	CMV infection more than 200 days after KT *	Adverse events	Discontinuation/dose reduction due to adverse events of VGCV	Acute rejection	eGFR at last follow-up (mL/min/1.73m^2^)
High (D + /R-)	1	666	Yes	No	FN^§^ (ANC 68/µL)	No	No	28.7^‡^
	2	599	Yes	Yes	FN^§^ (ANC 24/µL)	Discontinuation^†^	No	95.6
	3	440	No	Yes	FN^§^ (ANC 350/µL)	Dose reduction followed by discontinuation^†^	No	61.6
	4	431	Yes	No	Neutropenia^§^ (ANC 587/µL)	Dose reduction	No	77.3
	5	285	Yes	No	Neutropenia^§^ (ANC 779/µL)	No	No	85.3
Intermediate (R +)	6	507	No	No	Neutropenia^§^ (ANC 1,064/µL)	Dose reduction	No	149.4
	7	395	No	No	No	No	No	80.9
	8	371	No	No	No	No	No	85.3
	9	288	No	No	No	No	No	62.0

*ANC* absolute neutrophil count, *CMV* cytomegalovirus, *D* donor, *eGFR* estimated glomerular filtration rate, *FN* febrile neutropenia, *KT* kidney transplantation, *R* recipient, *VGCV* valganciclovir

^*^CMV disease was not observed in any patient with CMV infection

^†^VGCV was discontinued and subsequently resumed in these patients

^‡^The markedly reduced graft function observed in Patient 1 at the last follow-up was attributed to chronic T cell–mediated rejection related to medication non-adherence

^§^Antibiotics and granulocyte colony–stimulating factor (G-CSF) were administered in all cases of FN. In addition, Patients 4 and 5 received G-CSF for the management of neutropenia

To provide additional context for the timing of CMV-related events across protocol eras, protocol-specific cumulative incidence curves for CMV infection in high-risk recipients are shown in Supplementary Figure S1. In the first and second studies, CMV infection generally did not occur until approximately 200 days after KT, whereas in the third, fourth, and present studies, many patients developed CMV infection within 200 days. All high-risk recipients eventually developed CMV infection during the observational period, regardless of the regimen. No intermediate-risk patients developed CMV infection during the observational period.

### Adverse events of VGCV prophylaxis

In the present study, neutropenia occurred in six patients, five of whom were classified as high risk and one as intermediate risk (Table 2 and Fig. 1). Of these, three high-risk patients developed FN (Patients 1–3); one patient had severe neutropenia, whereas the remaining two had profound neutropenia. Dose reduction and/or discontinuation of VGCV prophylaxis due to AEs were required in four patients (Table 2 and Fig. 1). Among these, VGCV prophylaxis was discontinued in two patients because of FN (Table 2 and Fig. 1). In Patient 1, FN occurred not during VGCV prophylaxis but after GCV treatment for CMV infection. In Patient 5, VGCV prophylaxis was discontinued after seroconversion because the attending physician judged that the CMV infection had been adequately treated, rather than due to VGCV-related AEs.

Protocol-specific AEs-free rate across sequential studies are shown in Supplementary Figure S2, demonstrating that AEs occurred frequently in the first, second, and present studies, particularly under relatively higher VGCV dosing regimens.

In the present study, no episodes of acute rejection were observed during the observational period. The median eGFR at the last follow-up was 80.9 mL/min/1.73 m^2^ (IQR, 61.8–90.5 mL/min/1.73 m^2^). No clinically significant deterioration in allograft kidney function attributable to VGCV exposure was observed, except in one patient with chronic T cell–mediated rejection related to medication nonadherence.

## Discussion

In this single-center study, reduced-dose VGCV prophylaxis in pediatric patients after KT was associated with a high incidence of CMV infection within 200 days after KT in high-risk patients. Frequent hematologic AEs, including neutropenia and FN, were also observed and often resulted in VGCV dose reduction or discontinuation. These findings underscore the persistent difficulty of achieving an optimal balance between antiviral efficacy and safety in this population. However, no patients developed CMV disease, and all patients who experienced CMV infection subsequently achieved seroconversion. Therefore, reduced-dose VGCV prophylaxis may still represent an acceptable strategy for preventing CMV disease.

We compared the present study with our previous dose-optimization studies (Table 3). Because these studies were conducted under different protocols across distinct time periods, the comparisons should be interpreted descriptively. The first and second studies used VGCV at 51–90% and 26–67% of the recommended dose, respectively, and generally suppressed CMV infection within 200 days after KT but were associated with frequent hematologic AEs [6, 7]. In contrast, lower-dose regimens of 25–33% of the recommended dose were associated with a high incidence of CMV infection, suggesting insufficient antiviral efficacy [8, 9]. The present study evaluated an intermediate dosing strategy corresponding to 50% of the recommended VGCV dose. However, high-risk recipients continued to demonstrate frequent CMV infection and hematologic AEs, underscoring the persistent challenge of balancing antiviral efficacy and safety. In contrast, the 33% regimen remained acceptable in intermediate-risk patients, consistent with our previous observations.

**Table 3:** Summary of the present study and previous institutional dose-optimization studies

	First study^6)^	Second study^7)^	Third study^8)^	Fourth study^9)^	This study
Number of patients	5	7	4	12	9
High risk (D + /R-)	5	1	2	5	5
VGCV dose (the percentage of the recommended dose*)	450 mg daily (51–90%)	450 mg every other day (26–67%)	Half of the recommended dose every other day (25%)	One-third of the recommended dose daily (33%)	High: half of the recommended dose daily (50%) Intermediate: one-third of the recommended dose daily (33%)
Median observational period (days)	527	374	354	506	431
AEs^†^	4 (80%)	6 (86%)	0 (0%)	3 (25%)	6 (67%)
Discontinuation / Dose reduction due to AEs	3 (60%) / 1 (20%)	3 (43%) / 2 (29%)	0 (0%) / 0 (0%)	2 (17%) / 1 (8%)	2 (22%) / 3 (33%)
CMV infection occurring within 200 days following KT (Overall / High risk)	1 (20%) / 1/5 (20%)	0 (0%) / 0 (0%)	1 (25%) / 1/2 (50%)	4 (33%) / 3/5 (60%)	4 (44%) / 4/5 (80%)
CMV infection more than 200 days after KT (Overall / High risk)	4 (80%) / 4/5 (80%)	2 (29%) / 1/1 (100%)	1 (25%) / 1/2 (50%)	1 (8%) / 1/2 (50%)	2 (22%) / 2/5 (40%)

All studies summarized in this table were conducted in pediatric kidney transplant recipients at our institution. Low-risk recipients were not included in the dose-optimization analyses because antiviral prophylaxis was not routinely administered in this risk category

*AEs* adverse events, *CMV* cytomegalovirus, *D* donor, *KT* kidney transplantation, *R* recipient, *VGCV* valganciclovir

^*^The recommended dose was calculated using the U.S. Food and Drug Administration–recommended pediatric dosing formula: 7 × estimated glomerular filtration rate (eGFR; mL/min/1.73 m^2^) × body surface area (m^2^), expressed in milligrams[10]

^†^In the first study, AEs comprised anemia (n = 2) and neutropenia (n = 2); anemia was not observed in the subsequent studies, whereas neutropenia was observed across all studies

Several studies have described the efficacy of reduced-dose VGCV prophylaxis in adult and pediatric patients after KT. Gabardi et al. demonstrated that, among high-risk adult patients receiving VGCV prophylaxis after KT, administration of 50% of the recommended dose was not associated with a significantly higher incidence of CMV infection compared with the full recommended dose [17]. Pappo et al. reported that VGCV administered at a reduced, weight-based dose of 17 mg/kg, corresponding to approximately two-thirds of the recommended dose, was acceptable in terms of antiviral efficacy in pediatric kidney and liver transplant recipients, including both high-risk and intermediate-risk [18]. In contrast, in the present study, a high incidence of CMV infection within 200 days after KT was observed in high-risk pediatric patients treated with VGCV at 50% of the recommended dose. These findings suggest that higher VGCV exposure may be required for sufficient suppression of CMV infection in pediatric patients after KT. In the intermediate-risk group, an adult study comparing prophylactic and preemptive strategies after KT reported CMV infection rates of 46% in the prophylaxis group and 72% in the preemptive therapy group [19]. By contrast, no patients in the intermediate-risk group in the present study developed CMV infection, suggesting that the dosing regimen used in this study, corresponding to 33% of the recommended dose and consistent with our previous report, may provide sufficient efficacy in intermediate-risk patients [9].

VGCV-related AEs are common, making safety an important consideration. In pediatric patients receiving VGCV prophylaxis at 100% of the recommended dose for 200 days after KT, 39% of whom were classified as high risk, nearly half experienced leukopenia, neutropenia, or anemia, leading to treatment discontinuation in approximately 11% of cases [20]. Similarly, hematologic AEs, including severe cytopenias, have been reported with 100-day VGCV prophylaxis at the full recommended dose, leading to discontinuation in 6% of patients, in a cohort that included 26% high-risk recipients [21]. In the present study, all five high-risk patients developed neutropenia or FN despite the reduced-dose VGCV regimens, and VGCV prophylaxis was discontinued in two patients. Taken together with our previous reports [6, 7], these findings suggest that Japanese pediatric patients may be more susceptible to VGCV-related hematologic AEs, underscoring the need for careful monitoring. Notably, a polymorphism in NUDT15, which is more prevalent in East Asian populations, has been reported to be associated with VGCV-induced neutropenia [22]. This genetic background may partly explain the higher incidence of hematologic AEs observed in our cohort, although NUDT15 polymorphisms were not analyzed in our patients. Among intermediate-risk patients, neutropenia occurred in only one patient and resolved with VGCV dose reduction alone without treatment discontinuation, indicating that the reduced-dose regimen at 33% of the recommended dose was acceptable from a safety perspective in this group.

Despite dose stratification according to CMV risk, challenges remained in both antiviral efficacy and safety in the high-risk group. In this context, recent studies have shown that prophylaxis with letermovir, a CMV-specific terminase inhibitor, is non-inferior to VGCV in preventing CMV infection after KT, with a significantly lower incidence of neutropenia [23]. Given the high frequency of VGCV-related hematologic AEs observed in our cohort and previous reports [6, 7], switching to letermovir prophylaxis may be considered for high-risk pediatric patients after KT on a case-by-case basis.

This study has several limitations. The small sample size may limit the generalizability of our results to the broader pediatric patients after KT. Second, VGCV dose adjustments and the management of AEs were not strictly standardized and were determined based on routine clinical practice, which may have introduced variability in treatment approaches. In addition, potential confounding factors, including differences in immunosuppressive regimens and individual patient characteristics, could not be fully controlled. Therefore, larger multicenter studies are warranted to better define the optimal VGCV prophylaxis strategy in pediatric patients after KT.

## Conclusion

In the high-risk group, administration of VGCV at 50% of the recommended dose was associated with a high incidence of CMV infection within 200 days after KT, along with a high frequency of FN. Although no cases of CMV disease occurred and seroconversion was achieved in all high-risk patients, the overall efficacy and safety profile of the VGCV dosing regimen used in this study was suboptimal. By contrast, in the intermediate-risk group, the VGCV dosing regimen used in the present study appeared effective and tolerable. Further optimization of prophylactic strategies is required, particularly for high-risk pediatric recipients.

## Supplementary Information

Below is the link to the electronic supplementary material.

Supplementary file1 (PDF 198 KB)

## Data Availability

The datasets generated and/or analyzed during the current study are available from the corresponding author on reasonable request.
