# Supplementary material for: Efficacy and safety of reduced-dose valganciclovir prophylaxis for cytomegalovirus infection after pediatric kidney transplantation
Source: Clin Exp Nephrol. 2026 May 13;30(8):1245–52. doi: 10.1007/s10157-026-02876-z (PMC13379471; doi:10.1007/s10157-026-02876-z)
Supplement: Supplementary file 1 — Supplementary file1 (PDF 198 KB) [file 10157_2026_2876_MOESM1_ESM.pdf]

Supplementary Material

Supplements to: Ryo Nakatani, Yoko Shirai, Taro Ando, Yoko Yamasaki, Yohei Kume, Koji Tsugawa, Kohei Unagami, Tomokazu Shimizu, Hideki Ishida, Kenichiro Miura

Efficacy and safety of reduced-dose valganciclovir prophylaxis for cytomegalovirus infection after pediatric kidney transplantation

Supplementary Figure S1.

Protocol-specific CMV infection-free rate in high-risk recipients across sequential VGCV prophylaxis studies

Supplementary Figure S2.

Protocol-specific AEs-free rate in high-risk recipients across sequential VGCV prophylaxis studies

Supplementary Figure S1.

Protocol-specific CMV infection-free rate in high-risk recipients across sequential VGCV prophylaxis studies

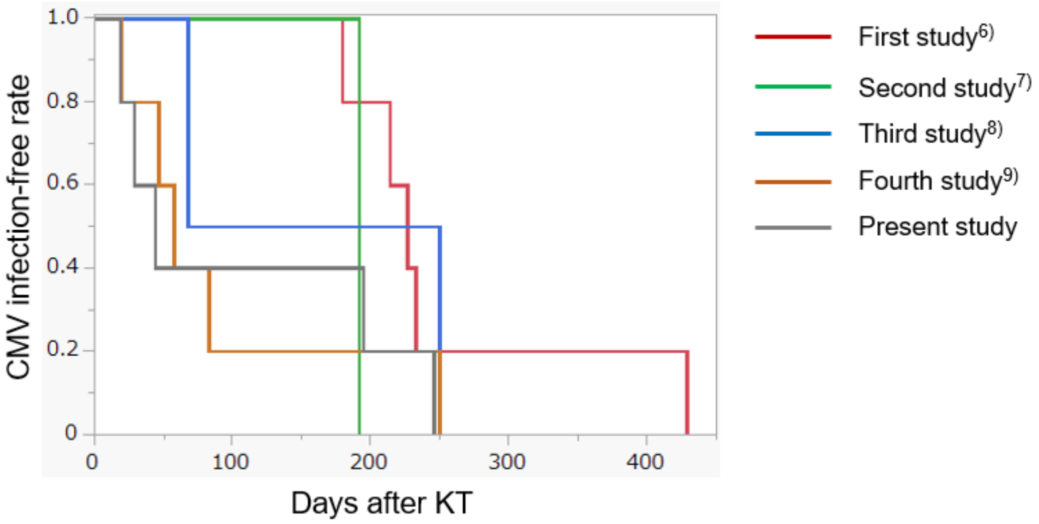

Number at risk

First study	5	5	4	1	1
Second study	1	1	0	0	0
Third study	2	1	1	0	0
Fourth study	5	1	1	0	0
Present study	5	1	1	0	0

Legend: Curves are presented for descriptive purposes only because studies were conducted during different clinical periods with evolving surveillance strategies. The VGCV dosing regimens were as follows: the first and second studies administered VGCV as a fixed 450-mg tablet dose, corresponding to 51–90% and 26–67% of the recommended dose, respectively; the third study used 25% of the recommended dose, and the fourth study used 33% of the recommended dose. The present study evaluated a regimen corresponding to 50% of the recommended dose. All patients experienced CMV infection within the observation period; therefore, no censoring occurred in this analysis. In the first and second studies, CMV infection was generally prevented until approximately 200 days after KT, whereas in the third, fourth, and present studies, many patients developed CMV infection within 200 days.

Supplementary Figure S2.

Protocol-specific AEs-free rate in high-risk recipients across sequential VGCV prophylaxis studies

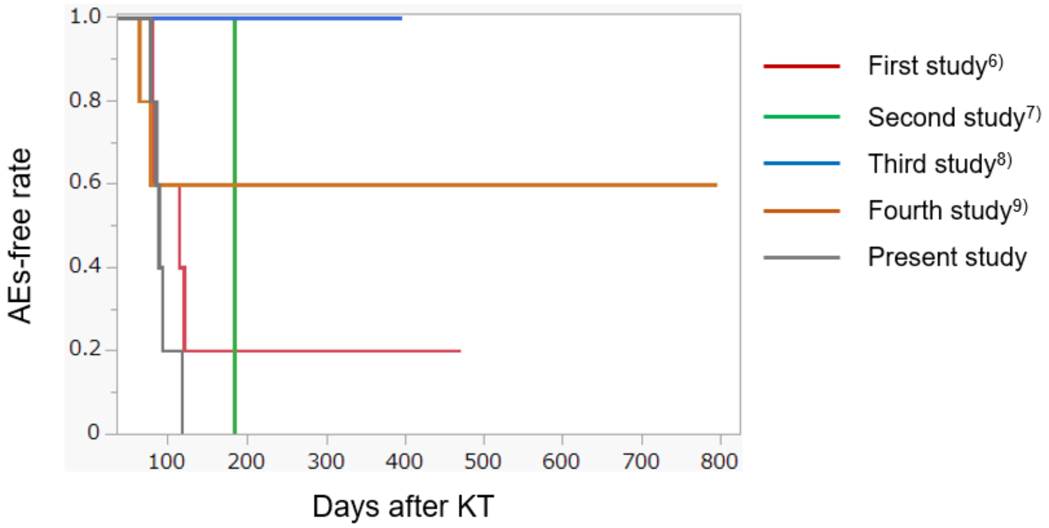

Number at risk

First study	5	3	3	0	0
Second study	1	0	0	0	0
Third study	2	2	0	0	0
Fourth study	5	3	3	3	0
Present study	5	0	0	0	0

Legend: In the first and second studies, AEs were frequent, whereas in the third and fourth studies, where lower doses of VGCV were used, AEs were relatively less common. In the present study, despite the intermediate regimen, AEs were observed in all cases.
